# Identification, Biological Activities and Biosynthetic Pathway of *Dendrobium* Alkaloids

**DOI:** 10.3389/fphar.2021.605994

**Published:** 2021-04-20

**Authors:** Zongmin Mou, Yi Zhao, Fei Ye, Yana Shi, Edward J. Kennelly, Suiyun Chen, Dake Zhao

**Affiliations:** ^1^ Biocontrol Engineering Research Center of Plant Disease and Pest, Biocontrol Engineering Research Center of Crop Disease and Pest, School of Ecology and Environmental Science, Yunnan University, Kunming, China; ^2^ Department of Biological Sciences, Lehman College and The Graduate Center, City University of New York, Bronx, NY, United States; ^3^ Ph.D. Programs in Biochemistry, Biology, and Chemistry, The Graduate Center, City University of New York, New York, NY, United States; ^4^ Kunming Municipal Hospital of Traditional Chinese Medicine, Kunming, China; ^5^ College of Agriculture and Biotechnology, Yunnan Agricultural University, Kunming, China; ^6^ Institute of Medicinal Plants, Yunnan Academy of Agricultural Sciences, Kunming, China

**Keywords:** alkaloids, orchidaceae, *Dendrobium*, anti-inflammatory, antitumor, mechanisms, biosynthetic pathway

## Abstract

*Dendrobium* is a genus of flowering plants belonging to the Orchidaceae family with more than 1,400 species. Many *Dendrobium* species have been used as medicinal plants in several Asian countries for thousands of years. Alkaloids were reported as the major biological markers due to their complex chemical compositions and various types. In this review, we summarized the structural types of alkaloids, their pharmacological activities, as well as the mechanisms of biological activities. More than sixty alkaloids were isolated and identified from the *Dendrobium* genus. Moreover, the pharmacological effects of *Dendrobium* alkaloids as hepatic lipid and gluconeogenesis regulation, as neuroprotection, and as anti-tumor, anti-inflammatory, anti-diabetes, and anti-virus factors were described. Besides, the total chemical synthesis of dendrobine is provided, while the biosynthetic pathway of dendrobine has been proposed based on the functions of associated genes. For applications of these invaluable herbs, more researches on the extraction of biological markers from compounds are needed. Further confirmation of the proposed biosynthetic pathways is anticipated as well.

## Introduction

Apart from Asteraceae, the orchid family is the second-largest flowering family, which has 28,000 species distributed in about 736 genera (Chase et al., 2015), among which *Dendrobium* is one of the largest genera. It contains more than 1,500 species (www.theplantlist.org), most of which are epiphytic or lithophytic, and it is widespread in South, East, and Southeast Asia, like China, Japan, Philippines, Vietnam, India, and Indonesia. Some species are also found in New Guinea, Australia, and the islands of the Pacific (Zhu et al., 2009). The plants of *Dendrobium* species have been used as traditional or folk medicine in many Asian countries for thousands of years. For instance, there are 96 *Dendrobium* species in China, and about 30 species, known as shí hú (石斛) or shí hú lán (石斛兰), have been widely used as ethnic medicine for tonifying the stomach, nourishing Yin (to enhance the production of body fluids, such as blood, saliva, tears, etc.), and clearing heat and toxic matter (Yang et al., 2006; Flora of China, 2020). The earliest written record of the medicinal usage of *Dendrobium* was found in the ancient text “Shen Nong’*s Herbal Classic*” 2000 years ago, in which it was considered to be a “superior grade” herbal medicine. Hundreds of years later, *Dendrobium* was documented in detail in “Compendium of Materia Medica” in Ming Dynasty (1590 AD). Nowadays, *Dendrobium nobile* Lindl., *Dendrobium chrysotoxum* Lindl., *Dendrobium fimbriatum* Hook., *Dendrobium officinale* Kimura et Migo, and *Dendrobium huoshanense* Z. Z. Tang et S. J. Cheng are included in Chinese Pharmacopoeia (2020 edition). Among these five species, *Dendrobium nobile* Lindl. is one of the 50 fundamental herbs used in traditional Chinese medicine (TCM). Generally, the fibrous stems of *Dendrobium* are employed as the officinal parts in ethnopharmacology, persevered by dry processing (Chinese Pharmacopoeia Commission, 2020). These stems are usually used either alone or mixed with other tonic Chinese herbs, like xī yáng shēn (American Ginseng) and gǒu qǐ zǐ (Barbary Wolferry Fruit) (Lin et al., 2018). Aside from China, *Dendrobium* species have also been used as ethnomedicines in Japan, Indian, and Thailand (Table 1). Given their high medicinal value and wide ethno-applications, the *Dendrobium* genus was recognized as a prized folk medicine (Ng et al., 2012).

**TABLE 1:** The ethnomedicine use of *Dendrobium* in some countries.

Country	Dendrobium species	Local name	Ethnomedicine use	References
China	*Dendrobium nobile* Lindl.	shí hú (石斛)	Used as a tonic and antipyretic for treating human disorders	Lam et al. (2015)
	*Dendrobium chrysotoxum* Lindl.	shí hú lán (石斛兰)	Used for clearing heat and toxic matter, and enhancing immunity	Lin et al. (2018)
	*Dendrobium fimbriatum* Hook.	huáng cǎo (黄草)		Yang et al. (2006)
	*Dendrobium officinale* Kimura et Migo	xiān cǎo (仙草)		Yuan et al. (2018)
	*Dendrobium aphyllum* (Roxb.) C.E.C.Fisch			Wang et al. (2020)
	*Dendrobium huoshanense* Z. Z. Tang et S. J. Cheng			
	*Dendrobium findlayanum* C. S. P. Parish et Rchb. f.			
	*Dendrobium loddigesii* Rolfe			
Japan	*Dendrobium moniliforme* (L.) Sw.	Fu-ran	Gives long life to men	Cakova et al. (2020)
Indian	*Dendrobium macraei* Lindl. (jeevanti)	Charaka samhita	Used as an astringent to the bowels, as anaphrodisiac, and in asthma and bronchitis	Lam et al. (2015)
	*Dendrobium alpestre* royle (jewanti)	Ayurved		
		Jeevanti		
Thailand	*Dendrobium draconis* Rchb. F.	Unknown	Employed as a blood tonic	Ng et al. (2012)

Due to the important pharmacological activities and economical value of *Dendrobium* genus, up to now, many phytochemical and pharmacological researches have been implemented. The active constituents in *Dendrobium* are polysaccharides, alkaloids, flavonoids, amino acids, bibenzyls, and several trace elements (He et al., 2020). The polysaccharides from *Dendrobium* exhibit immunomodulatory and hepatoprotective activities; and the alkaloids are antioxidant, anticancer, and neuroprotective, while other compounds display anti-angiogenesis, anti-cytotoxicity, and anti-mutagenesis effects (Ng et al., 2012; Xu et al., 2013). Alkaloids are the earliest identified category of compounds in *Dendrobium* (Chen and Chen, 1935). More importantly, *Dendrobium* alkaloids are the key constituents that responsible for their pharmacological activities, making them potential candidates for new drugs. Therefore, some important bioactive markers such as dendrobine (20) have attracted many scientists to investigate their chemical, pharmaceutical, and biological mechanisms, as well as biogenetic pathways (Li Q. et al., 2017).

*Dendrobium* alkaloids with complex chemical structures consist of pyrrole, indolizidine, terpenoid alkaloids, organic amine alkaloids, indole, quinazoline, and others (Xu et al., 2013). In accordance with other genera of Orchidaceae plants, indolizidine alkaloids and organic amine alkaloids are the major constituents of this genus (Lam et al., 2015). These chemicals are considered as active ingredients for effects like anti-inflammatory, cytotoxic, antitumor, cytoprotection, gluconeogenesis regulation, and preventing neuronal apoptosis (Ng et al., 2012). For instance, *Dendrobium nobile* Lindl. is a famous TCM recorded in Chinese Pharmacopoeia (2020 edition). The alkaloids of *Dendrobium nobile* Lindl. (DNLA) are considered to have beneficial effects on liver metabolism, hepatic lipid homeostasis, neuronal activity, and resistance effects on tumors, cancers, and virus based on previous studies (Table 2). Dendrobine (20), a sesquiterpene alkaloid, makes up 92.6% of the DNLA (Xu et al., 2017). Dendrobine (20) is the first identified active alkaloid of *Dendrobium nobile* Lindl. (Chen and Chen, 1935), and is regarded as the standard agent for qualitative and quantitative evaluation of *Dendrobium nobile* Lindl. (Li R. et al., 2017).

**TABLE 2:** Summary of the pharmacological of alkaloids isolated from *Dendrobium nobile* Lindl.

No	Organ	Alkaloids content of crude extract	Constituents of alkaloid extract	Pharmacological activities	References
1	Stem	79.8%	Dendrobine (20), 92.6%	Beneficial effects on liver glucose and lipid metabolism gene expressions	Xu et al. (2017)
			Dendrobine-N-oxide (22), 3.3%		Li et al. (2019)
			Nobilonine (45), 2.0%		
			Dendroxine (24), 0.9%		
			6-Hydroxy-nobilonine (46), 0.32%		
			13-Hydroxy-14-oxodendrobine (26), 0.07%	Protective effects on CCl_4_-induced acute liver injury	
2	Stem	79.8%	Dendrobine (20), 92.6%	Protective effects on hepatic lipid homeostasis and acute liver injury	Huang S. et al. (2019); Zhou et al. (2020)
3	Stem	Unkown	Mixed fat-soluble alkaloids	Anti-tumor efficacy in human colorectal cancer	He et al. (2017)
4	Unkown	96.1%	Dendrobine (20), 90.7%	Protection from OGD/RP-induced neuronal damages	Wang et al. (2010)
			Dendramine (23), 2.31%		
			3-Hydroxy-2-oxodendrobine (26), 1.29%		
			Nobilonine, (45), 4.47%		
5	Stem	96.1%	Dendrobine (20), 90.7%	Protection of brain impairment	Li et al. (2011)
			Nobilonine (45), 4.47%		
			Dendramine (23), 2.31%		
			3-Hydroxy-2-oxodendrobine (26), 1.29%		
6	Unkown	54.5%	Dendrobine (20), 30.5%	Attenuation of LPS-induced hyperphosphorylation of tau protein and protection against LPS-induced apoptosis	Yang et al. (2014)
7	Unkown	79.8%	Dendrobine (20)	Prevention of neuronal apoptosis and synaptic loss	Nie et al. (2016)
			Dendrobine-N-oxide (22)		
			Nobilonine (45)	Regulation of a- and ß-secretase in hippocampal neurons	Huang J. et al. (2019)
			Dendroxine (24)		
			6-Hydroxy-nobilonine (46)		
			13-Hydroxy-14-oxodendrobine (26)		
8	Dendrobine standard	>98%	Dendrobine (20), >98%	Anticancer activity toward non-small cell lung cancer cells	Song et al. (2019)
9	Dendrobine standard	98%	Dendrobine (20), 98%	Anti-influenza a virus	Li et al. (2017)

In this review, we aim to summarize the structural types, pharmacological activities, and the mechanisms of biological activities of *Dendrobium* alkaloids. Additionally, the proposed biogenetic pathways of dendrobine (20) are also included.

## Structural Identification of *Dendrobium* Alkaloids

**FIGURE 1:**
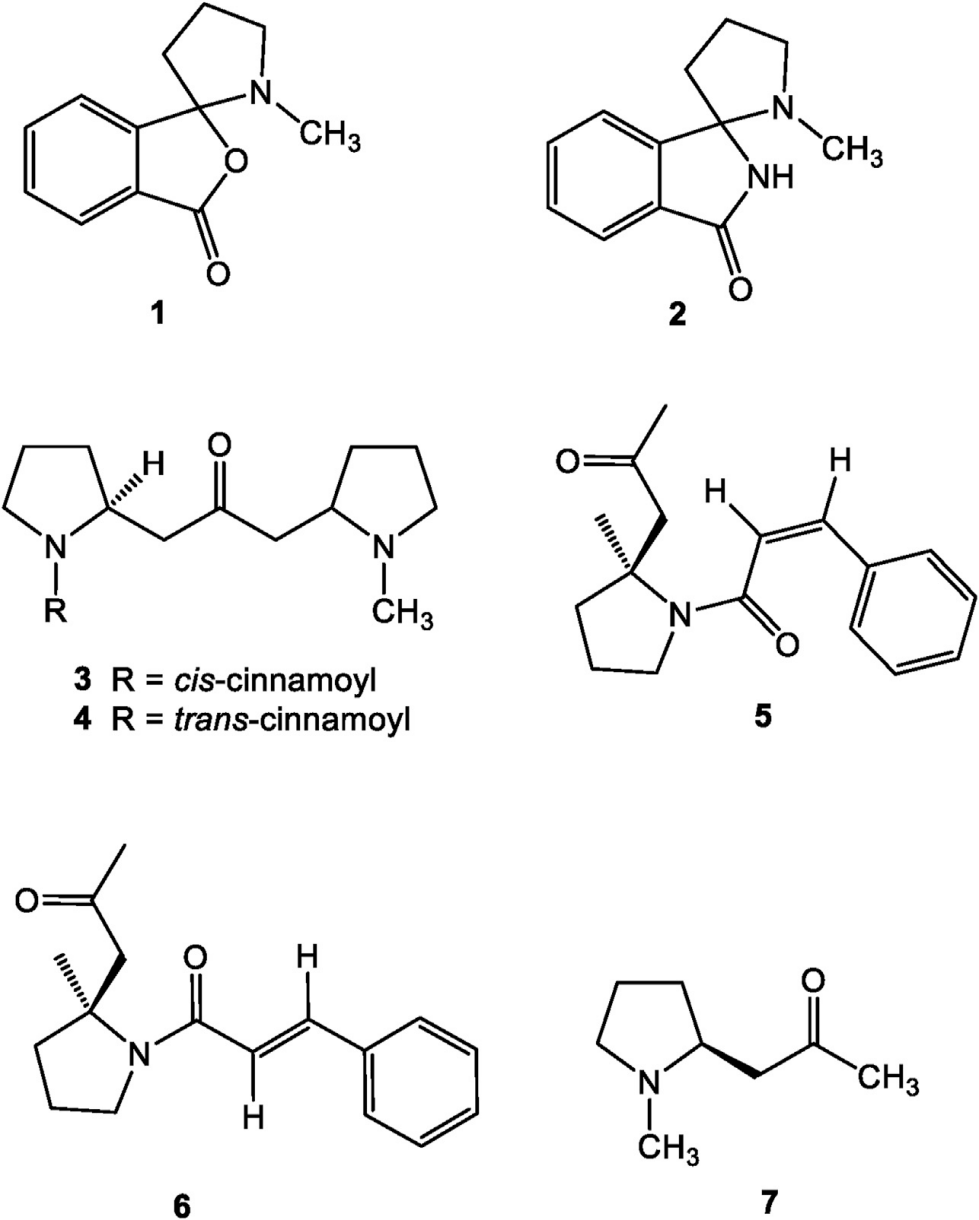
Structures of pyrrole alkaloids reported in *Dendrobium*.

**FIGURE 2:**
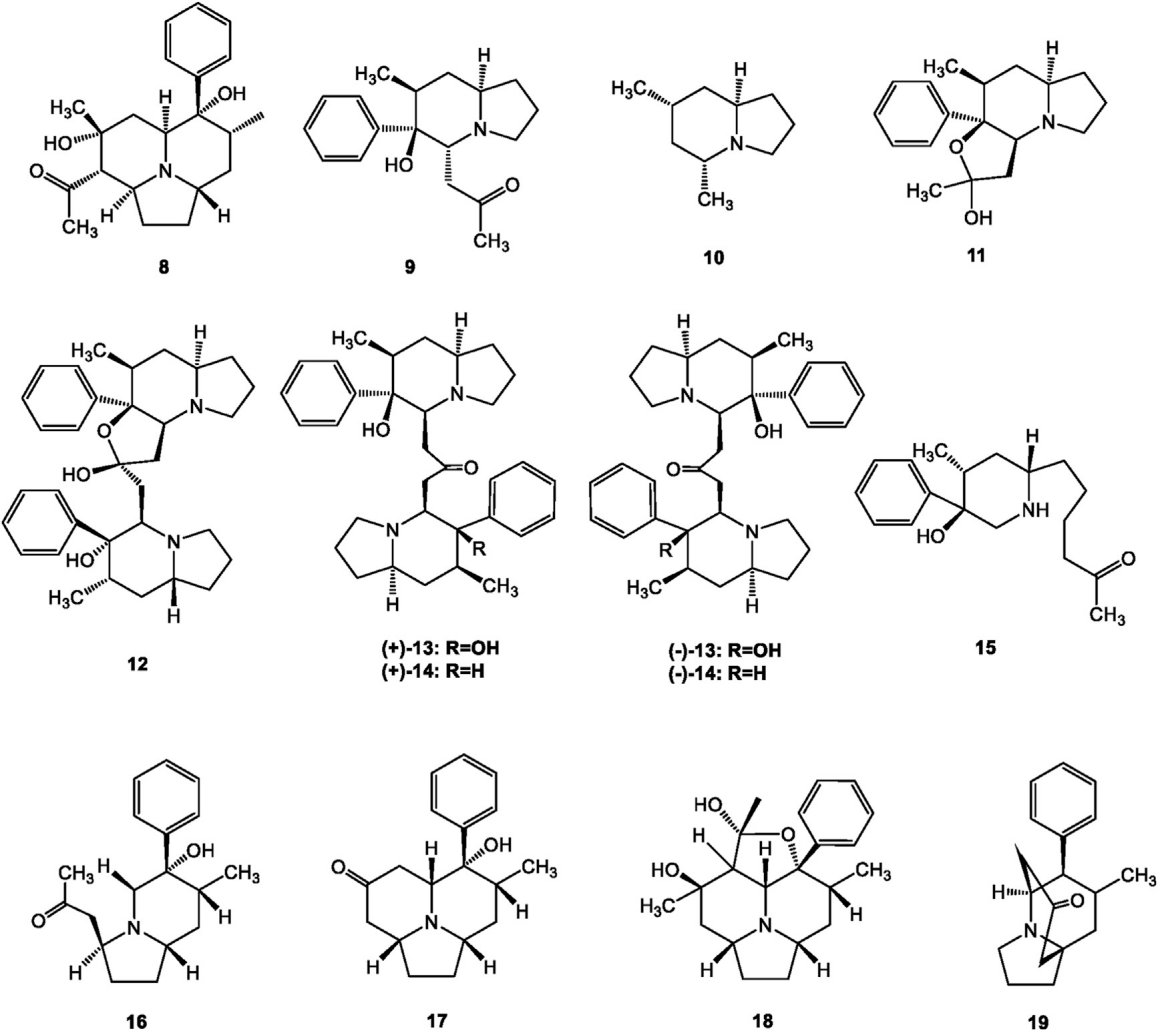
Structures of indolizidine alkaloids reported in *Dendrobium*.

**FIGURE 3:**
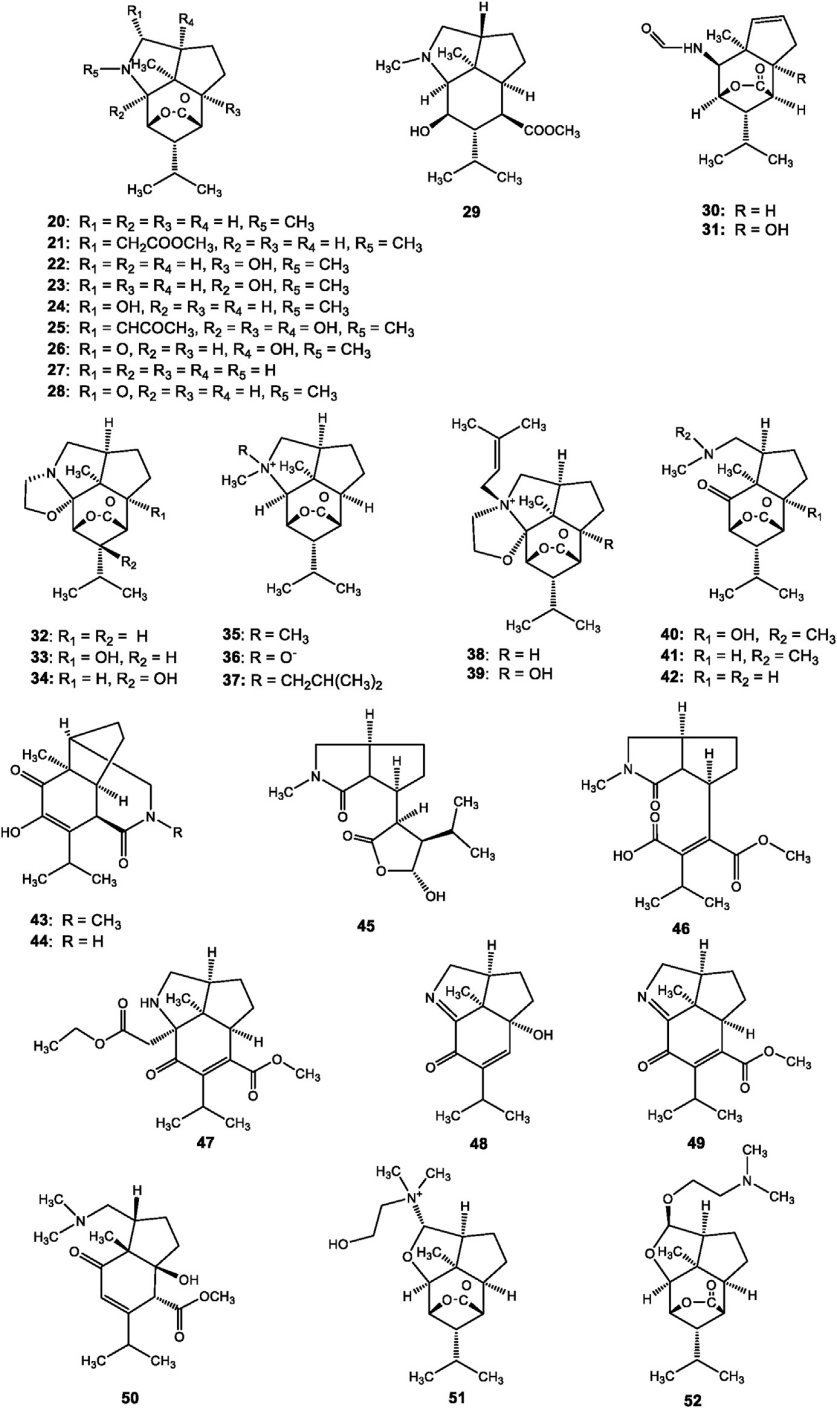
Structures of terpenoid alkaloids reported in *Dendrobium*.

**FIGURE 4:**
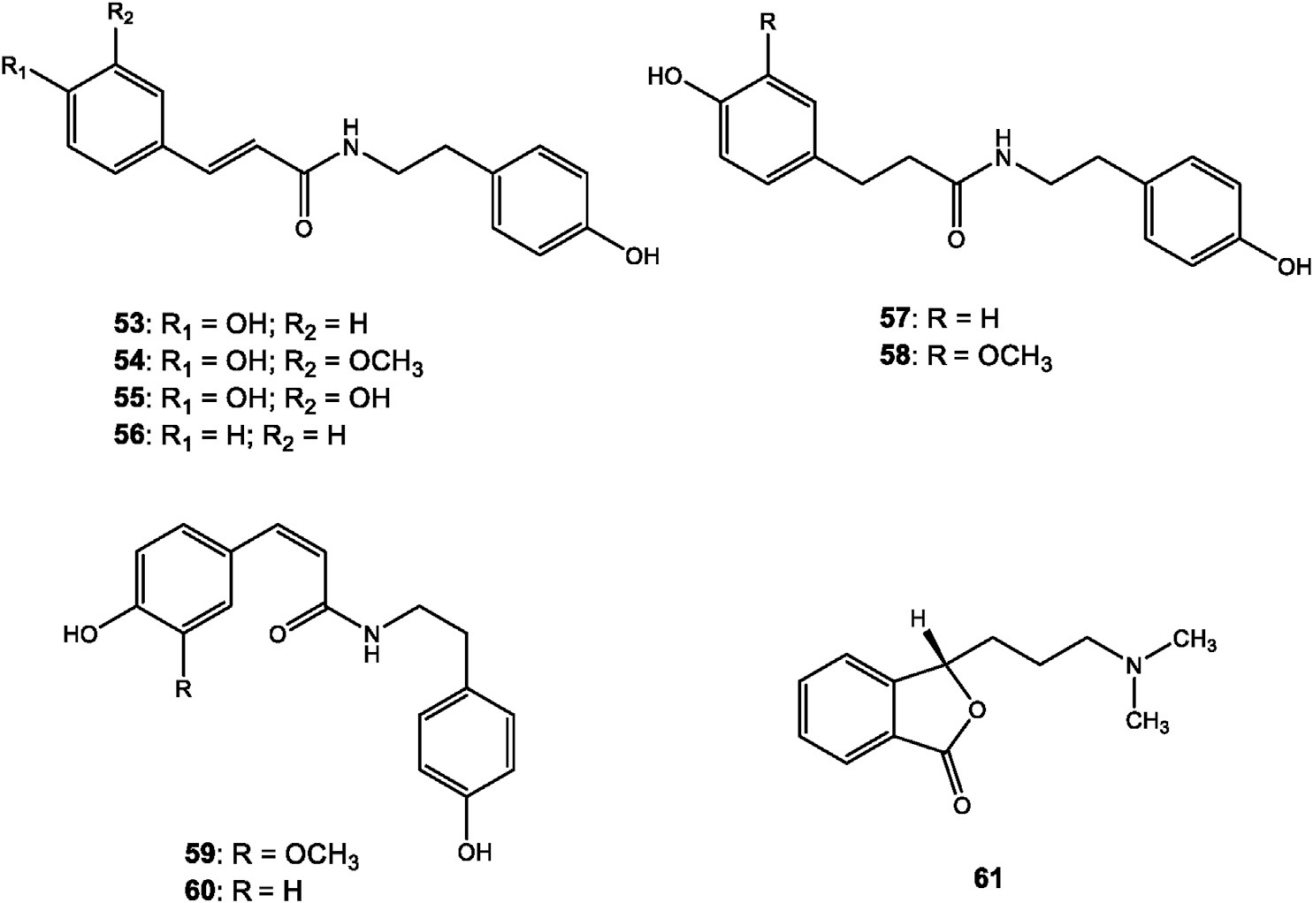
Structures of organic amine alkaloids reported in *Dendrobium*.

**FIGURE 5:**
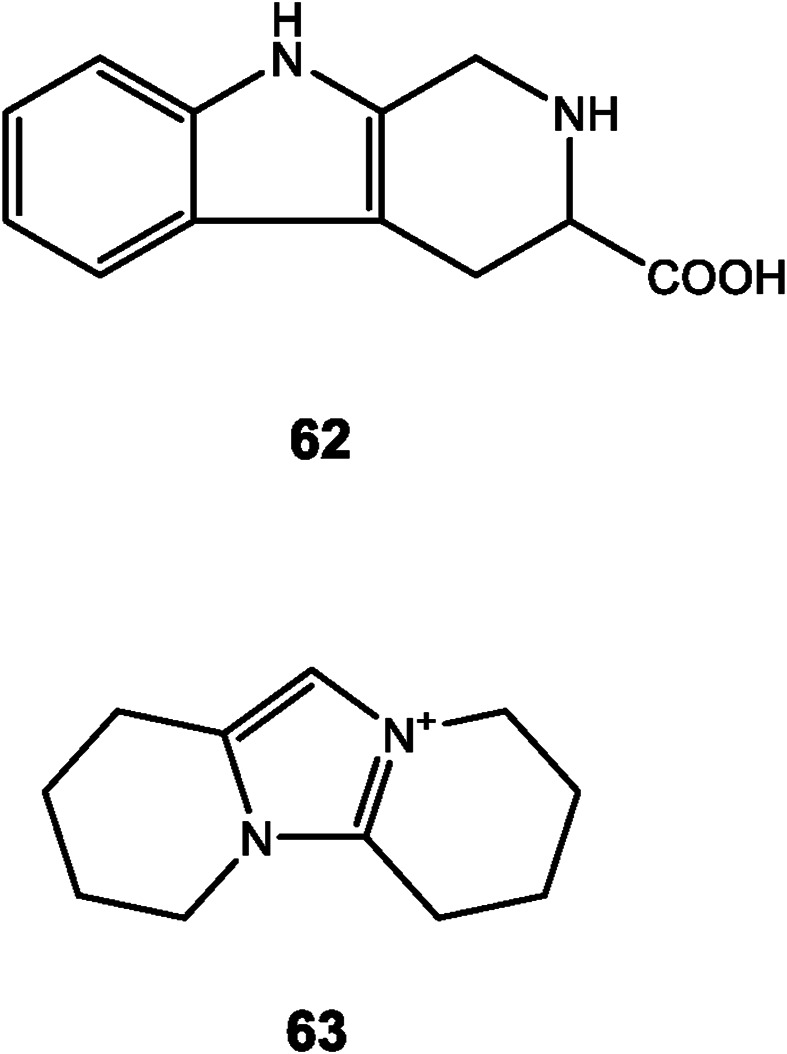
Structures of indole and other alkaloids reported in *Dendrobium*.

**FIGURE 6:**
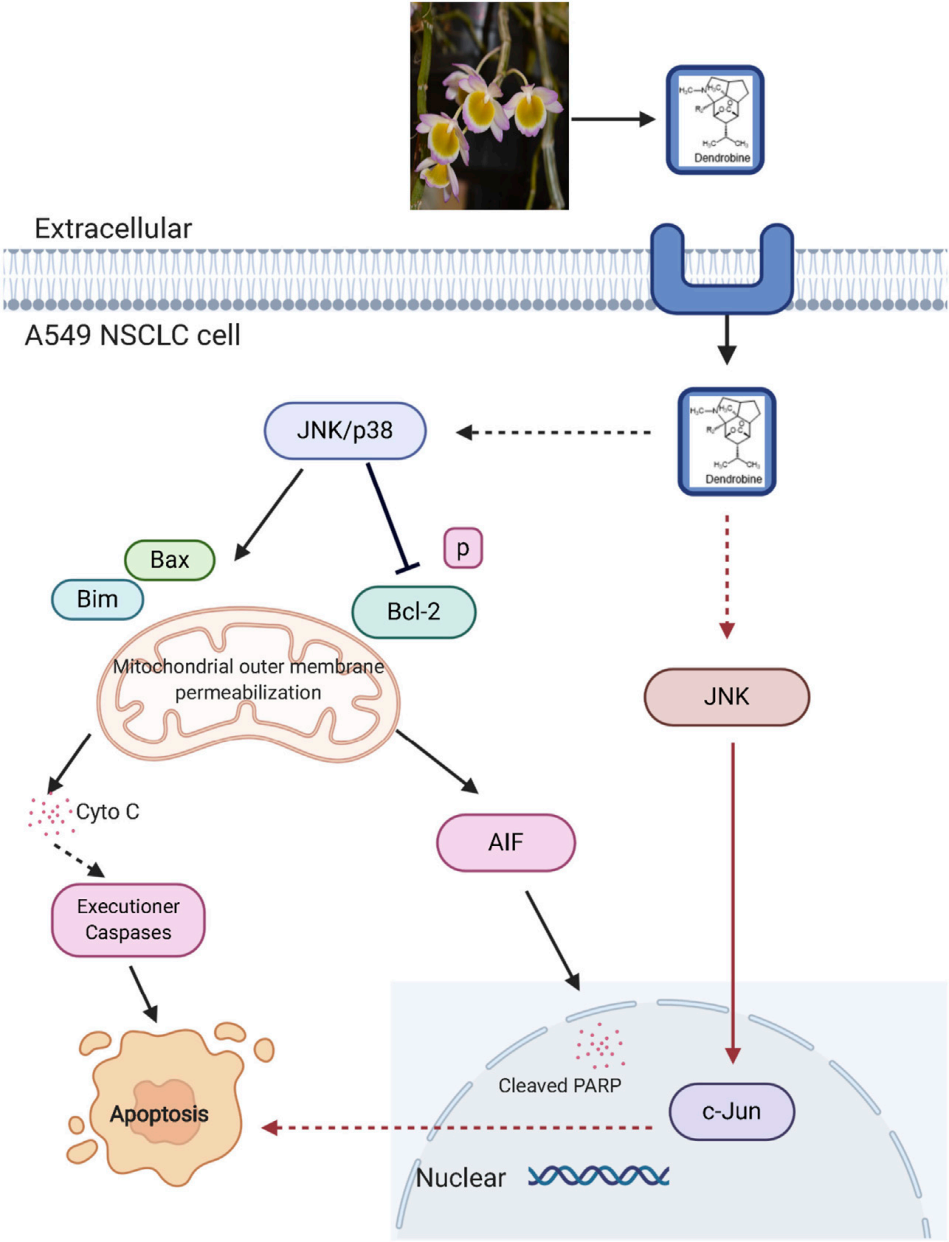
Signaling pathway involved in dendrobine induced apoptosis in cancer cells. JNK, c-Jun N-terminal kinase; p38, p38 mitogenactivated protein kinase; Cyto C, cytochrome C; AIF, apoptosis inducing factor; Blc-2, B-cell lymphoma two; Bim, Bcl-2 interacting mediator of cell death; PARP, poly ADP-ribose polymerase.

**FIGURE 7:**
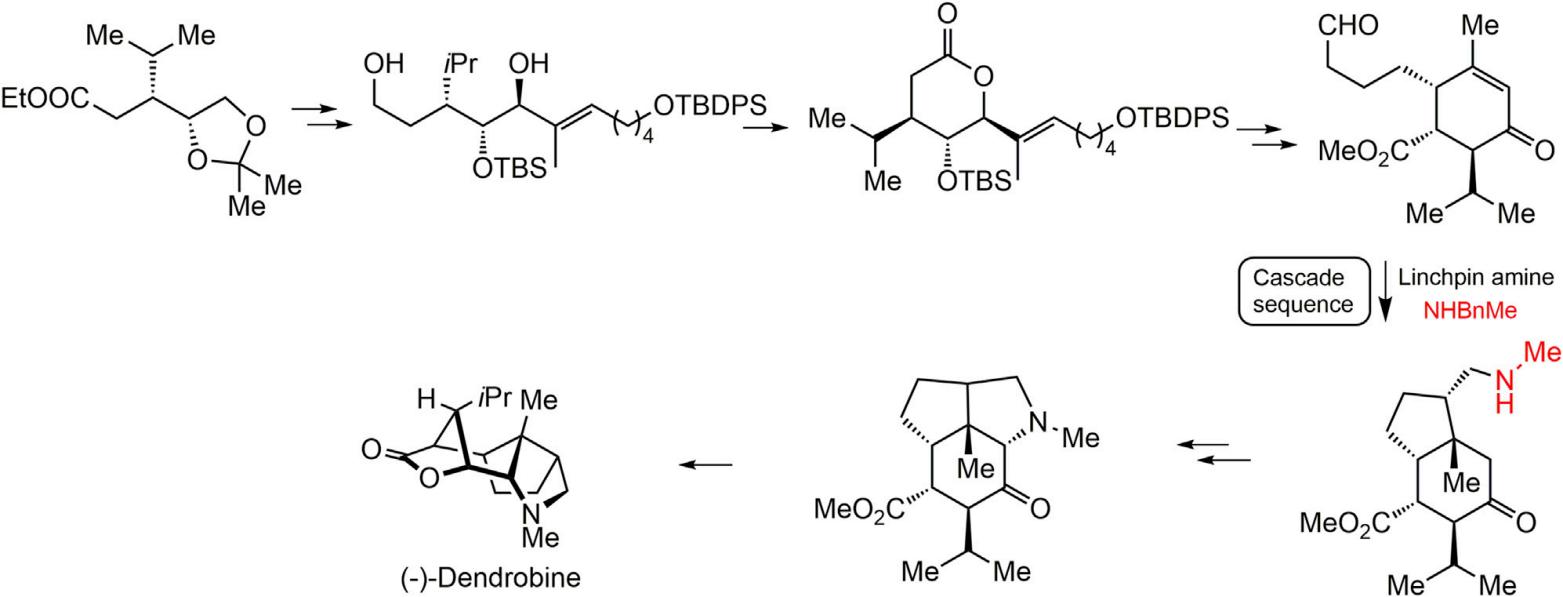
The total chemical synthesis strategy of (-)-dendrobine proposed by Kries and Carreira (2012).

**FIGURE 8:**
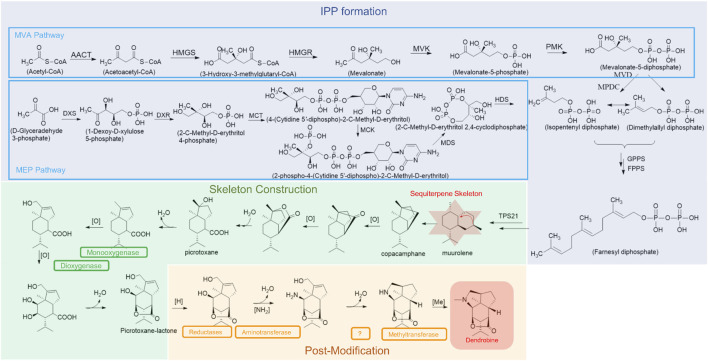
The potential biosynthetic pathway of dendrobine.

Alkaloids are representatives of the first category of compounds extracted from *Dendrobium* (Xu et al., 2013). *Dendrobium* alkaloids were isolated by the traditional alkaloid extraction method given their basic chemical structure. Dried powders of *Dendrobium* spp. were liquid-liquid extracted with various solvents, such as ethanol, methanol, or chloroform, then fractionated successively with water, petroleum ether, ethyl acetate, n-butyl alcohol, etc. (Yang et al., 2020). Subsequently, these fractions were purified on different silica gel column chromatography systems with various polarity ranges of solvents (Morita et al., 2000). Moreover, high performance liquid chromatography (HPLC) and ultra-performance liquid chromatography (UPLC) coupled with mass spectrometry were developed to discover new compounds of *Dendrobium* (Xu et al., 2020). Up to now, more than sixty alkaloids (Figures 1–5, 1–63) have been identified from this genus. The chemical structures include pyrrole, indolizidine, terpenoid, amine, and indole alkaloids. These compounds were mainly isolated from the whole plants, stems, or leaves of *Dendrobium nobile* Lindl., *Dendrobium officinale* Kimura et Migo, *Dendrobium findlayanum* C. S. P. Parish et Rchb. f., *Dendrobium chrysanthum* Wall. ex Lindl., *Dendrobium crepidatum* Lindl. ex Paxton, *Dendrobium anosmum* Lindl., *Dendrobium devonianum* Paxton, *Dendrobium friedericksianum* Rchb. f., *Dendrobium hildebrandii* Rolfe, *Dendrobium loddigesii* Rolfe, *Dendrobium lohohense* Tang et F. T. Wang, *Dendrobium moniliforme* (L.) Sw., *Dendrobium pierardii* R. Br., *Dendrobium primulinum* Lindl., and *Dendrobium wardianum* R. Warner (Inubushi et al., 1964; Lüning et al., 1965; Elander et al., 1969; Ekevag et al., 1973; Begum et al., 2010; Lam et al., 2015; Hu et al., 2016; Hu et al., 2016; Xu et al., 2019).

### Pyrrole Alkaloids

Most pyrrole alkaloids from Orchidaceae were found in *Dendrobium*, *Pleione*, and *Liparis* plants. Till now, only five pyrroles were reported in *Dendrobium*, and all of them are simple phthalide-pyrrolidine alkaloids (Figure 1). Shihunine (1), a water-soluble phthalide-type alkaloid, was the first pyrrole alkaloid from *Dendrobium lohohense* Tang et F. T. Wang in 1968 (Inubushi et al., 1964). Shihunidine (2) was also isolated from the same species by Li et al. (1991). Cis-trans isomerizations of dendrochrysines (3 and 4) and dendrochrysanines (5 and 6), the other four pyrrole isomers alkaloids, were isolated from *Dendrobium chrysanthum* Wall. ex Lindl. (Ekevag et al., 1973; Yang et al., 2005), while hygrine (7) was produced in *Dendrobium primulinum* Lindl. (Lüning et al., 1965).

### Indolizidine Alkaloids

Indolizidine alkaloids are important constituents of *Dendrobium* (Xu et al., 2019). Twelve indolizidine alkaloids were observed in *Dendrobium*, most of which were from *Dendrobium crepidatum* Lindl. et Paxton (Figure 2, 8–19). Dendroprimine (8) is a simple indolizidine alkaloid reported in *Dendrobium primulinum* Lindl. (Lüning et al., 1965). Other indolizidine alkaloids such as crepidine (9), crepidamine (10), isocrepidamine (11), and isodendrocrepine (12) were found in *Dendrobium crepidatum* Lindl. et Paxton (Elander et al., 1973; Hu et al., 2020). The other three alkaloids of this type (±)-homocrepidine A [(±)-13] (±)-dendrocrepidamine A [(±)-14], and homocrepidine B (15) were first identified from the same *Dendrobium* species by Hu et al. (2016; 2020). Then the absolute configurations of the new pairs enantiomeric octahydroindolizine compounds (±)-homocrepodine A [(±)-13] and (±)- dendrocrepidamine A [(±)-14], were verified by single-crystal X-ray diffraction (Hu et al., 2016; Hu et al., 2020). Recently, four new indolizidine alkaloids, crepidatumines A to D (16–19), were purified from *Dendrobium crepidatum* Lindl. et Paxton by Xu et al. (2019; 2020).

### Terpenoid Alkaloids

Terpenoid alkaloids are another important secondary metabolites principally isolated from *Dendrobium* (Xu et al., 2013). The types of alkaloids are various based on their mono-, sesqui-, di-, and tri-terpenoid skeletons. Dendrobine (20) was the first terpenoid-alkaloid elucidated from *Dendrobium nobile* Lindl. in 1932 (Chen and Chen 1935). Subsequently, a total of 25 dendrobine-type alkaloids were found in *Dendrobium nobile* Lindl., *Dendrobium findlayanum* C. S. P. Parish et Rchb. f., *Dendrobium wardianum* R. Warner, and *Dendrobium moniliforme* (L.) Sw., most of which are sesquiterpenoid alkaloids (Figure 3, 20–42, 51–52). Interestingly, dendrobine-type alkaloids are a class of characteristic picrotoxanes with highly complex structures, which are only distributed in *Dendrobium* genus (Begum et al., 2010; Meng et al., 2017). All of these dendrobine-type alkaloids contain basic skeletons comprising one picrotoxane-type sesquiterpenoid combined with a five-membered C2-C9-lined N-heterocycle and C3-C5-linked lactonic ring (Xu et al., 2013).

Two thirds of terpenoid alkaloids in the genus *Dendrobium* were isolated from the certain species of *Dendrobium nobile* Lindl.. Mubironines A-C (27–29) were identified from the whole plant (Morita et al., 2000), and the absolute components of these three compounds were confirmed by single-crystal X-ray diffraction. Dendroterpene A and B (30–31) were found from the stems recently (Wang P. et al., 2019). Other terpenoid alkaloids from *Dendrobium nobile* Lindl., compounds 20–26 and 32–41 have been reported over eighty years (Chen and Chen, 1935; Shhosuke and Yoshimasa, 1964; Inubushi and Nakano, 1965; Okamoto et al., 1966a; Okamoto et al., 1966b; Granelli et al., 1970; Elander and Leander, 1971; Hedman and Leander, 1972; Okamoto et al., 1972; Glomqvist et al., 1973; Wang et al., 1985; Meng et al., 2017; Wang Q. et al., 2019; Liu et al., 2020; Yang et al., 2020). A total of eleven terpenoid alkaloids were purified from another three *Dendrobium* species. Dendrofindline B (42) was isolated from *Dendrobium findlayanum* C. S. P. Parish et Rchb. f. (Liu et al., 2020). Besides, seven new seco-dendrobines, findlayines A-F (43–48), and dendrofindline A (49) were identified from the same species. (Yang et al., 2018; Liu et al., 2020; Yang et al., 2020). In 2007, Liu et al. (2007) reported the isolation and structural identification of moniline (50) from the stems and leaves of *Dendrobium moniliforme* (L.) Sw.. Dendrowardine (51) and wardianumine A (52) were purified from *Dendrobium wardianum* R. Warner by Liu and Zhao. (2003) and Zhang et al. 2017, respectively.

### Amine Alkaloids

Amine alkaloids are a class of widely spread natural amines with basic nitrogen but cannot form a ring in the skeleton. Most amines in *Dendrobium* are amides (Figure 4, 53–61). For example, *N-cis*-p-coumaroyltyramine (53) and *N-cis*-feruloyltyramine (54) were identified from the stems of *Dendrobium devonianum* Paxton (Zhang et al., 2013). Pierardine (61) was isolated from *Dendrobium pierardii* R. Br. (Elander et al., 1969).

### Indole and Other Types of Alkaloids

2,3,4,9-tetrahydro-1 H-pyrido [3,4-b] indole-3-carboxylic acid (62) was the only reported indole alkaloid from *Dendrobium devonianum* Paxton (Zhang et al., 2013) (Figure 5). Moreover, anosmines (63) are another type of alkaloids isolated from two species of *Dendrobium*, whose structures were confirmed by X-ray crystallography (Hemscheidt and Spenser, 1993).

### Metabolic Analysis of *Dendrobium*

At present, metabolomics has been widely utilized in the field of medicinal plants, such as bioactive components identification, drug metabolism, toxicology, and investigation on metabolic pathways, etc. (Liang et al., 2009). Alkaloids are regarded as chemical markers in quantitative analysis of *Dendrobium*. Generally, the metabolic profiling of *Dendrobium* alkaloid compounds was established by liquid chromatography coupled to single (LC-MS) and tandem (LC-MS/MS) mass spectrometry, in combination with multivariate data analyses, where secondary metabolites can be accurately quantified based on their fingerprint chromatograms. For example, the comparative metabolite analysis of *Dendrobium officinale* Kimura et Migo and *Dendrobium huoshanense* Z. Z. Tang et S. J. Cheng showed that the accumulation of alkaloids was species-specific (Song et al., 2020). Ten potential anti-inflammatory alkaloid components were detected from the extraction of *Dendrobium aphyllum* (Roxb.) C. E. C. Fisch by UPLC-MS (Wang P. et al.,2019), while eight water-soluble metabolites containing rare imidazolium alkaloids and anosmines (4) were identified by the screening of *Dendrobium nobile* Lindl., *Dendrobium officinale* Kimura et Migo, and *Dendrobium loddigesii* Rolfe, using chromatography along with spectroscopic techniques (Chen et al., 2018). Besides, DNLA was reported to improve hepatic lipid homeostasis based on the results of UPLC-MS of 48 kinds of hepatic bile acids in the livers of high fat diet (HFD)-fed mice (Huang S. et al., 2019). Furthermore, the combination of metabolomic and transcriptomic technologies revealed the possible pathways in alkaloid biosynthesis of *Dendrobium officinale* Kimura et Migo (Guo et al., 2013).

## Pharmacological Activities

*Dendrobium* alkaloids are active components with anti-inflammatory, antitumor, and anti-viral effects, which can also regulate hepatic lipid and gluconeogenesis, and protect from hyperglycemia. For a better understanding of the bioactivities of *Dendrobium* alkaloids, previous studies on pharmacological efficacy are summarized.

### Anti-inflammatory Activity

Inflammation induced by endotoxin such as lipopolysaccharide (LPS), is an immune defense response of organisms to tissue injury and microbial agents (Guha and Mackman, 2001). Most anti-inflammatory activities were tested with the LPS-induced RAW264.7 model by evaluating the indices of nitric oxide (NO) production and the expression of inducible NO synthase (Chen et al., 2018). The anti-inflammatory activities of *Dendrobium* alkaloids have been reported. For example, anosmines (63) that were presented in four *Dendrobium* species exhibited inhibitory activity against NO production and inflammation in LPS-activated RAW264.7 cells without cytotoxic activity (Chen et al., 2018). Besides (+)-homocrepidine A [(+)-13] isolated from *Dendrobium crepidatum* Lindl. ex Paxton was evaluated for its anti-inflammatory activity (NO inhibition) with LPS-induced RAW 264.7 macrophages, and the half maximal inhibitory concentration (IC_50_) value was 3.6 µM. However, the other enantiomeric isomer (–)-homocrepodine A [(–)-13], displayed an IC_50_ value of 22.8 µM, which was almost 7 times less active than [(+)-13]. Besides, their racemic mixtures (±)-homocrepodine A [(±)-13], showed a moderate inhibitory effect (IC_50_ = 5.0 µM). Similar pharmacological activities were observed in (±)-dendrocrepidamine A [(±)-14] (Hu et al., 2020). Compared with the enantiomers of racemic indolizidine and their racemic mixtures, homocrepidine B (15) also displayed moderate anti-inflammatory activity with the IC_50_ value of 27.6 µM (Hu et al., 2016). Furthermore, the total alkaloids, mainly consisted of six indolizine-type compounds from the same *Dendrobium* species, showed protective effects against the LPS-induced acute lung injury in mice by the down-regulation of the TLR4-mediated MyD88/MAPK signaling pathway (Hu et al., 2018). Taken together, the pharmacological investigations of *Dendrobium* alkaloids on anti-inflammatory shed light on scientific guidance for the source of this genus.

### Improved Regulation of Hepatic Lipid Homeostasis and Gluconeogenesis

The liver is quite essential to the regulation of lipid and glucose homeostasis. On the other hand, the disruption of homeostasis will result in metabolic disorders of the liver, including fatty liver and diabetes, which are the most common chronic liver disease all over the world (Rinella, 2015). DNLA was found to impact the regulation of liver glucose and the expressions of lipid metabolism genes in mice livers by increasing the expressions of *PGC1a, Glut2, Cpt1a, Acox, ATGL/Pnpla2*, and *FoxO1* genes, and decreasing the mRNA transcription from the *Srebp1* gene (Xu et al., 2017). Moreover, excessive accumulation of hepatic lipids is responsible for liver metabolic dysfunction. Modulation of bile acids has been reported as an effective intervention strategy for maintaining hepatic lipid homeostasis (Chiang, 2013). DNLA exerted protective effects on hepatic lipid homeostasis by enhancing taurine-conjugated bile acids and decreasing the cholic acid/chenodeoxycholic acid ratio (Huang S. et al., 2019). To be specific, DNLA decreased four types of bile acids and increased five types of bile acids among 48 kinds of hepatic bile acids in the livers of high-fat diet (HFD)-fed mice (Huang S. et al., 2019). On the other hand, DNLA regulated hepatic gluconeogenesis by mediating the hepatic antioxidant components through hepatic metallothionein and the gene expression of the nuclear factor erythroid 2-related factor 2 antioxidant pathway, which plays critical roles in host defense against abnormal gluconeogenesis (Xu et al., 2017). The mechanisms were further elucidated that DNLA improved mitochondrial function and inhibited mitochondrial apoptotic cell death (Zhou et al., 2020). Overall, considering the beneficial effects of *Dendrobium* alkaloids on liver metabolism, *Dendrobium* alkaloids could be used as natural compounds in the development of new treatments for hyperlipidemia and hyperglycaemia.

### Anti-tumor Activity

It was reported that *Dendrobium* alkaloids could inhibit tumor cell growth and mediate apoptosis. (He et al., 2017; Song et al., 2019; Wang et al., 2014). Specifically, the alkaloid extracts of *Dendrobium candidum* Wall. ex Lindl. were reported to significantly inhibit the growth of transplanted Lewis tumors, meanwhile, the mixed alkaloids could improve the spleen index and regulate the expressions of TNF-α and IL-2 (Wang et al., 2014). Moreover, the fat-soluble alkaloids extracted from *Dendrobium nobile* Lindl. were found to induce the apoptosis of human colorectal cancer HT-29 cells with an IC_50_ value of 0.72 mg/ml at 48 h, where the cell cycle was arrested in G2 phase. Besides, the extraction decreased the mitochondrial membrane potential (ΔΨm) and induced ROS accumulation by increasing expression levels of apoptotic proteins, such as Caspase-9, Caspase-3, and intracellular cytochrome C (He et al., 2017), which may be related to the mitochondria-mediated apoptotic pathway. The combined treatment using sesquiterpene alkaloids, dendrobine (20) and cisplatin, was also effective for inhibiting the non-small cell lung cancer cells (NSCLC) *in vitro* and *in vivo*, where the cytotoxicity was induced by the simulation of c-jun NH_2_-terminal kinase (JNK)/p38 stress signaling pathways, and the expression change of pro-apoptotic proteins Bax and Bim further led to the apoptosis (Figure 6, revised from song et al., 2019, created with BioRender.com). Besides, dendrobine (20) also mediated apoptotic cell death by the mitochondrial-mediated pathway (Song et al., 2019). On the whole, due to the distinct association with cell death signaling pathways, dendrobine (20) can be regarded as a potential agent for the development of novel anti-NSCLS strategies especially when combined with cisplatin. (Song et al., 2019).

### Renal Protective and Anti-Diabetic Effects

In China, the dried stems of some *Dendrobium* species including *Dendrobium huoshanense* Z. Z. Tang et S. J. Cheng, *Dendrobium officinale* Kimura et Migo, and *Dendrobium nobile* Lindl. have been used to nourish kidney and improve the symptoms of diabetes (Cakova et al., 2020). For instance, shihunidine (2) and shihunine (1) isolated from *Dendrobium loddigesii* Rolfe displayed inhibitory effects on Na^+^/K^+^-ATPase of the rat kidney (Li et al., 1991). Li et al. (2019) recently reported that DNLA showed vital hypoglycemic effects in diabetic rats. The shihunine (1) extracts from *Dendrobium loddigesii* Rolfe at the dose of 50 mg/kg decreased the triglycerides level by 43.7%, compared with the non-treated db/db mice, and inhibited the expression of cleaved cysteine aspartic acid-specific protease 3. The result of western blot analysis also verified the agonistic effects of shihunine (1) extracts on the expressions of adenosine monophosphate-activated protein kinase phosphorylation and glucose transporter four in the liver or adipose tissues. Moreover, in clinical application, *Dendrobium* combined with other herbs, such as *Astragalus* spp. and *Schisandra* Michx., was applied for the therapy of diabetes (Cakova et al., 2020).

### Neuro-Protective Activity

It was reported that *Dendrobium* alkaloids exerted beneficial effects on neuronal systems (Wang et al., 2010; Li et al., 2011), among which *Dendrobium nobile* Lindl. was most extensively studied on the treatment of central nervous system disorders. DNLA, containing dendrobine (20), dendrobine-N-oxide (22), nobilonine (45), dendroxine (24), 6-hydroxy-nobilonine (46), and 3-hydroxy-2-oxodendrobine (also referred as 13-hydroxy-14-oxoHudendrobine) (26), was known as the active components of *Dendrobium nobile* Lindl. (Wang et al., 2010; Xu et al., 2017; Nie et al., 2018). Investigation on the mechanisms underlying the neuroprotective effects of DNLA revealed that DNLA prominently improved the neurobehavioral performance and prevented LPS-induced elevation in tumor necrosis factor receptor one via inhibition of phosphorylated p38 mitogen-activated protein kinases and the downstream nuclear factor kappa-B signal pathway (Li et al., 2011; Ng et al., 2012). Moreover, DNLA decreased the level of intracellular amyloid β peptide (Aβ) by improving impaired autolysosomal proteolysis in amyloid precursor protein/presenilin one mice (Nie et al., 2018), and regulating α- and β-secretase in hippocampal neurons of Sprague-Dawley rats (Huang J. et al., 2019). The reduction of Aβ attenuated Aβ_25–35_-induced spatial learning and memory impairments by increasing the protein expression of neurotrophic factors, such as brain-derived neurotrophic factor, ciliary neurotrophic factor, and glial cell line-derived neurotrophic factor (Nie et al., 2016; Nie et al., 2018). Furthermore, DNLA lowered the LPS-induced hyperphosphorylation of tau protein and prevented neuronal apoptosis in rat brains (Yang et al., 2014). Given the neuro-protective effect of *Dendrobium* alkaloids, they could be promising therapeutic agents for the treatment of neurodegenerative disorders, such as Alzheimer’s disease (Cakova et al., 2020).

### Anti-influenza A Virus Activity

Dendrobine (20) displayed antiviral activity against influenza A viruses, including A/FM-1/1/47 (H1N1), A/Puerto Rico/8/34 H274Y (H1N1), and A/Aichi/2/68 (H3N2) in the antiviral assay, plaque assay, time-of-addition assay, and pseudovirus neutralization assay, with IC_50_ values of 3.39 ± 0.32, 2.16 ± 0.91, and 5.32 ± 1.68 μg/ml, respectively. The low IC_50_ values of dendrobine (20) indicated that this compound could be applied as potential promising agents to treat influenza virus infection (Li R. et al., 2017). More importantly, the anti-virus test using dendrobine (20) provided valuable information for the full application of the TCM named “shí hú” (Li R. et al., 2017).

## Chemical Synthesis and Biosynthetic Pathway of Dendrobine

Dendrobine (20) is the first identified sesquiterpene alkaloid from *Dendrobium nobile* Lindl., which is recommended as the exclusive chemical marker for the quality control of this species by Chinese Pharmacopoeia (2015 and 2020 edition). The rule suggested that the mass fraction of dendrobine (20) should be greater than 0.4% in the medicinal *Dendrobium nobile* Lindl..

### Chemical Synthesis of Dendrobine

Dendrobine (20) with a complicated tetracyclic ring system and seven contiguous stereocenters displayed remarkable bioactivities. Up to now, several cases are available on the total chemical synthesis of dendrobine (20). Connolly and Heathcock (1985) first synthesized dendrobine (20) in 1985. Several decades later, Kreis and Carreira (2012) achieved the total chemical synthesis based on 18 cascaded reactions with a key amine group, and the main synthesis pathway is summarized in Figure 7 (Kreis and Carreira, 2012). Other three dendrobine-alkaloids (–)-dendrobine (20) (–)-mubironine B (27), and (–)-dendroxine (24) were also obtained by total synthesis (Guo et al., 2018). Despite the advances of these total synthesis methods, it remains challenging to overcome the compound yield after a series of reactions (Li Q. et al., 2017).

### Biosynthesis of Dendrobine

Dendrobine (20) belongs to the class of terpenoid indole alkaloids (TIAs) (Wang et al., 2020). The biogenetic pathway of TIAs is conservative among alkaloid-producing plants (Li Q. et al., 2017). Based on the results of transcriptome and metabolomic analysis, the putative dendrobine (20) biosynthetic pathway was proposed, and a series of key metabolic genes were labeled in Figure 8 (Guo et al., 2013; Li Q. et al., 2017; Chen et al., 2019).

Three core stages were involved in the biogenetic pathway, including the formation of isopentenyl diphosphate (IPP), the construction of sesquiterpene skeleton, and the process of post-modification. Firstly, the mevalonate (MVA) and 2-C-methyl-D-erythritol 4-phosphate (MEP) pathways were considered as the upstream of dendrobine (20) biosynthetic pathway, mainly for the synthesis of IPP (Chen et al., 2019). Three key enzyme-coding genes involved in the MVA pathway, *acetyl-CoA C-acetyltransferase (AACT)* gene, *phosphomevalonate kinase (PMK)* gene, and *diphosphomevalonate decarboxylase (MVD)* gene, were observed to be positively associated with dendrobine (20) accumulation in *Dendrobium nobile* Lindl. through large-scale transcriptome sequencing, and then validated through qRT-PCR analysis (Li Q. et al., 2017). In contrast, *hydroxymethylglutaryl-CoA synthase (HMGS)* gene and *3-hydroxy-3-methylglutaryl coenzyme A reductase (HMGR)* gene were found to be less effective in dendrobine (20) biosynthesis in the same species (Li Q. et al., 2017), though *HMGS* and *HMGR* both played significant roles in alkaloid biosynthesis in *Dendrobium officinale* Kimura et Migo (Chen et al., 2019). The result shows that *HMGS* and *HMGR* may differently contribute to the production of dendrobine (20) in *Dendrobium* spp.. In the MEP pathway, rate-determining genes *1-deoxy-d-xylulose-5-phosphate synthase (DXS)* and *1-deoxy-d-xylulose-5-phosphate reductoisomerase (DXR)* isolated from protocorms of *Dendrobium officinale* Kimura et Migo were largely up-regulated by the methyl jasmonate (MeJA) treatment, suggesting their significant roles in the sesquiterpene biosynthesis based on the analysis of KEGG enrichment and relative expression (Fan et al., 2016; Chen et al., 2019). The crucial impacts of *DXS* and *DXR* in *Dendrobium officinale* Kimura et Migo were later confirmed by the high correlations between total alkaloid contents and their transcripts (Chen et al., 2019), furthermore, *DXS* was a leaf-specific expression gene accounting for high alkaloids content in leaves (Shen et al., 2017).

IPP is an important downstream product of MVA and MEP pathways, which is the precursor for the construction of synthetic terpenes. IPP formed the skeleton of muurolene-type sesquiterpene initially catalyzed by TPS21 enzyme (Li Q. et al., 2017), then this sesquiterpene was further oxidized by monooxygenases and/or dioxygenase to produce picrotoxane-lactone. Cytochromes P450s (CYP450s) is a complex superfamily of monooxygenase, and they are vital for the formation of sesquiterpene alkaloids (dendrobine). At present, some CYP450s have been discovered in a few *Dendrobium* species (Coon, 2005; Guo et al., 2013; Li Q. et al., 2017; Yuan et al., 2018; Chen et al., 2019). For instance, 59 full-length CYP450s candidate genes involved in the dendrobine (20) biosynthesis were identified and characterized in *Dendrobium officinale* Kimura et Migo through tissue-specific transcriptomic analysis, phylogenetic analysis, and further gene expression pattern induced by MeJA treatment (Chen et al., 2019). In *Dendrobium huoshanense* Z. Z. Tang et S. J. Cheng, 229 genes were identified as putative CYP450s, 7.8% of which were CYP71 family members associated with hydroxylation steps of alkaloid biosynthesis (Yuan et al., 2018). However, the family members and expression patterns of CYP450s remain unclear in most *Dendrobium* plants. It is worth mentioning that all other 25 dendrobine-type alkaloids (20–42, 51–52) identified from *Dendrobium* were believed to share similar biosynthesis pathways due to the mutual sesquiterpene backbone of these alkaloids (Guo et al., 2013; Chen et al., 2019).

Following the generation of sesquiterpene skeleton, dendrobine (20) was finally synthesized by the post-modification of picrotoxane-lactone with a series of enzymes, including reductases, aminotransferases, and methyltransferases (Guo et al., 2013; Yuan et al., 2018). In *Dendrobium nobile* Lindl., the expression level of *methyltransferase-like protein 23 (METTL23)* gene, *histone-lysine N-methyltransferase ATX4 (ATX4)* gene, and *alanine aminotransferase 2 (AAT2)* gene were enhanced after inoculation with MF23 (*Mycena* sp.), which was positively related with the content of dendrobine (20), implying their important roles in dendrobine (20) biosynthesis (Li Q. et al., 2017). Transcription factors play vital roles in modulating the expression of dendrobine (20) biosynthesis genes, such as C3H, bHLH, bZIP, MYB, and WRKY in *Dendrobium officinale* Kimura et Migo (Yuan et al., 2018).

Although the common biosynthesis pathway for most TIAs through the construction of strictosidine backbone exists in many plants (Wang et al., 2018), no enzyme involved in strictosidine formation has been verified in dendrobine (20) biosynthesis. However, due to the complex dendrobine (20) metabolism, accurate identification of genetic networks from a large number of candidate genes is needed in the future.

The metabolism of dendrobine (20) was affected by abiotic and biotic stresses. For example, light intensity was reported to influence the content of dendrobine (20) (Li J. L. et al., 2017). MeJA, a signaling molecule in the biosynthesis of alkaloids, could induce the accumulation of *Dendrobium* alkaloids by an active precursor supply (Chen et al., 2019). Besides, symbiosis with mycorrhizal fungus could stimulate the biosynthesis of dendrobine (20) by regulating the expressions of genes involved in the MVA pathway (Li Q. et al., 2017). Other relevant factors need to be further elucidated.

## Conclusion

In this paper, we summarized the structural types, pharmacological activities, and mechanisms of *Dendrobium* alkaloids, as well as the suggested biogenetic pathway of dendrobine (20), which is an important type of sesquiterpene alkaloids. Despite the advances of the investigation on alkaloids, more emphasis should be laid on the discovery of more novel skeletons in *Dendrobium* genus based on abundant alkaloid metabolites, and the improvement of isolation methods. Moreover, many current studies on *Dendrobium* were only focused on their crude extracts, or the activity of mixtures, which necessitates the need for figuring out the typical pharmacological activity of pure *Dendrobium* alkaloids. Additionally, further investigation on novel pharmacological activities of these alkaloids should be implemented. Meanwhile, in-depth researches on the biological mechanisms of these activities are also desired. Finally, although the biosynthetic pathway of dendrobine (20) has been proposed, further confirmation is anticipated.
